# Reasoning About Life Transitions in Relation to Perceived Housing: In-Depth Data From Germany

**DOI:** 10.1093/geroni/igab046.1718

**Published:** 2021-12-17

**Authors:** Anna Wanka, Karla Wazinski, Frank Oswald

**Affiliations:** 1 Goethe University Frankfurt, Frankfurt, Hessen, Germany; 2 Goethe University Frankfurt, Frankfurt am Main, Hessen, Germany

## Abstract

Partially different to the Swedish contribution, this paper analyses the relationships between perceived housing, life course transitions and wellbeing among community-dwelling older adults in Germany. Based on 15 qualitative interviews with persons aged 60-75 years, the contribution focuses on the experience of interrelationships between different life course transitions and perceived housing, and how they contribute to wellbeing in later life. First findings indicate a concourse of different transitions around the retirement age (e.g. illnesses, changes in partnerships) and a temporal as well as causal relationship between the two transitions relocation and retirement (for example, relocation becomes possible only after retiring or people relocate with the retirement phase in mind). The entanglement of life course transitions, in turn, shapes the person-place-relationships and perceived housing in different ways, which will be exemplified and interpreted in the presentation. However, further research is needed to consider the effects of social inequalities in these processes.

